# Cloning and Characterization of the Autoinducer Synthase Gene from Lipid-Degrading Bacterium *Cedecea neteri*

**DOI:** 10.3389/fmicb.2017.00072

**Published:** 2017-01-31

**Authors:** Kian-Hin Tan, Kah-Yan How, Jia-Yi Tan, Wai-Fong Yin, Kok-Gan Chan

**Affiliations:** Division of Genetic and Molecular Biology, Institute of Biological Sciences, Faculty of Science, University of MalayaKuala Lumpur, Malaysia

**Keywords:** *Cedecea neteri*, quorum sensing, cloning, LuxI homolog, AHL synthase, *N*-acyl homoserine lactone

## Abstract

The process of intercellular communication among bacteria, termed quorum sensing (QS), is mediated by small diffusible molecules known as the autoinducers. QS allows the population to react to the change of cell density in unison, in processes such as biofilm formation, plasmid conjugation, virulence, motility and root nodulation. In Gram-negative proteobacteria, *N*-acyl homoserine lactone (AHL) is the common “language” to coordinate gene expression. This signaling molecule is usually synthesized by LuxI-type proteins. We have previously discovered that a rare bacterium, *Cedecea neteri*, exhibits AHL-type QS activity. With information generated from genome sequencing, we have identified the *luxIR* gene pair responsible for AHL-type QS and named it *cneIR*. In this study, we have cloned and expressed the 636 bp *luxI* homolog in an *Escherichia coli* host for further characterization. Our findings show that *E. coli* harboring *cneI* produced the same AHL profile as the wild type *C. neteri*, with the synthesis of AHL known as *N*-butyryl-homoserine lactone. This 25 kDa LuxI homolog shares high similarity with other AHL synthases from closely related species. This work is the first documentation of molecular cloning and characterization of *luxI* homolog from *C. neteri*.

## Introduction

Bacteria communicate with each other through a phenomenon called quorum sensing (QS), which enables them to detect the cellular density of the population, thus directing proper genotypic and phenotypic adaptations accordingly (Bassler and Losick, 2006). This is achieved through the synthesis, release, detection, and response to small diffusible signaling molecules called the autoinducers (Schauder and Bassler, 2001). A diverse range of phenotypes has been discovered to be regulated by QS, such as biofilm formation, plasmid conjugation, virulence, motility and root nodulation (Williams et al., 2007).

*N*-acyl homoserine lactone (AHL) is the first autoinducer being studied and to date, it is also the most commonly studied type of autoinducer (Eberhard et al., 1981). The structure of AHL molecules varies, hence, providing signal specificity. However, all of them possess an invariant homoserine lactone ring. The length of the acyl side chain ranging from four to eighteen carbon atoms, the saturation level of the side chain, as well as the substitution group at the third carbon atom by oxo or hydroxyl group contribute to the variation in AHL structures (Whitehead et al., 2001; Williams et al., 2007).

Synthesis of AHL relies on the enzymatic activity of LuxI-type protein, which was first isolated from *Aliivibrio fischeri* (Engebrecht and Silverman, 1984). This enzyme, in *A. fischeri*, catalyzes the formation of *N*-(3-oxohexanoyl)homoserine lactone (OC6-HSL) and hexanoyl homoserine lactone (C6-HSL) using *S*-adenosylmethionine (SAM) and an acylated acyl carrier protein (ACP) from fatty acid biosynthesis pathway. SAM first binds to the active site of LuxI protein, and the acyl group from acylated ACP is then transferred to this protein. LuxI then catalyzes the formation of an amide bond between the acyl group with the amino group of SAM. Final lactonization results in the production of AHL and the by-product, 5′-methylthioadenosine. The affinity of the LuxI protein to specific acylated ACP gives rise to the product specificity of the enzyme. Interestingly, some LuxI homologs can react with more than one type of acylated ACP, resulting in the synthesis of more than one type of AHL molecules (Schaefer et al., 1996; Parsek et al., 1999).

Similar to other members of the genus *Cedecea, Cedecea neteri* is lipase positive and resistant to cephalothin and colistin but it does not hydrolyze gelatin or DNA. It differs from other species of *Cedecea* in that *C. neteri* is negative for ornithine decarboxylase (Moeller’s), fermentation of raffinose and melibiose, but positive for fermentation of sucrose, D-sorbitol, D-xylose, and malonate utilization, and it grows in media without thiamine. Originally known as “*Cedecea* species 4,” its name was coined when its role as a human pathogen was recognized in 1982 (Farmer et al., 1982). It was 13 years later that another case of bacteremia caused by *C. neteri* was reported. As these cases involved old and immunocompromised patients, *C. neteri* is posed as an opportunistic pathogen (Aguilera et al., 1995). However, the incidence of its isolation is infrequent and its role in human disease is mostly not well understood.

*C. neteri* has also been isolated from non-human sources such as vegetables and insects (Jang and Nishijima, 1990; Osterblad et al., 1999). In the previous study, we have reported the isolation of a strain of *C. neteri*, SSMD04, from pickled mackerel sashimi (Chan et al., 2014). There are several characteristics of this bacterium that drew our attention. First of all, it is capable of degrading lipid and thus, presenting potential biotechnological uses (Berman, 2012; Tan et al., 2015). Studies have shown that lipid-degrading microorganisms could be exploited as additive in detergent, lipid modification in the food industry and removal of the pitch from pulp in paper making (Sharma et al., 2001). This phenotype in strain SSMD04, coupled with its isolation from a food source, makes it a potential food spoilage agent. However, this speculation requires further evidence. Nevertheless, limited knowledge on this bacterium makes it an interesting subject of study. Apart from this, *C. neteri* was reported to exhibit resistance toward a wide range of antimicrobial agents, such as amoxicillin, cephalosporin, aminoglycosides, ampicillin, and others (Dalamaga et al., 2008; Mawardi et al., 2010; Abate et al., 2011). In fact, examination of the genome of strain SSMD04 using Rapid Annotations using Subsystems Technology (RAST) reveals multiple antimicrobial resistance genes toward fluoroquinolones, fosfomycin and β-lactam antibiotics (Chan et al., 2014). Despite all the characteristics discussed, it was when this bacterium exhibits QS activity that spiked our interest for further study as bacterial pathogenicity has been shown to be associated with QS (Rutherford and Bassler, 2012).

In our previous work, we demonstrated that *C. neteri* SSMD04 exhibits QS activity using biosensors and thus, hypothesized that QS potentially plays a role in the regulation of its virulence and lipolytic pathways. A pair of *luxIR* homolog was identified from its genome sequences (Tan et al., 2015). In this study, we have cloned the *luxI* homolog, *cneI*, and subjected to overexpression in *Escherichia coli* host and verified that this gene is responsible for the synthesis of the signaling molecule, *N*-butyryl-homoserine lactone (C4-HSL). The current work facilitates the understanding of the QS mechanism in the bacterium, thus shedding light into its biology.

## Materials and Methods

### Bacterial Strains and Culture Conditions

*C. neteri* SSMD04 was isolated from *shime saba* as previously described (Tan et al., 2015). It was grown aerobically in a Luria-Bertani (LB) (Merck, Germany) medium at 28°C with shaking (220 rpm). Propagation of recombinant plasmids and overexpression of fusion protein were performed using *E. coli* DH5α (Invitrogen, USA) and BL21(DE3)pLysS (Novagen, Germany), respectively, in which the strains were cultured in LB medium at 37°C with shaking. If necessary, the bacterial cultures were supplemented with 100 μg/ml ampicillin (Sigma, St. Louis, MO, USA), 30 μg/ml kanamycin (Sigma, St. Louis, MO, USA) or 34 μg/ml chloramphenicol (Sigma, St. Louis, MO, USA).

### Nucleic Acids Extraction

One milliliter of an overnight culture of SSMD04 was centrifuged to collect the cell pellet and genomic DNA was extracted using MasterPure DNA Purification Kit (Epicentre, Inc., Madison, WI, USA) whereas plasmids were extracted using QIAprep Spin Miniprep Kit (Qiagen, Germany) according to manufacturer’s protocol. NanoDrop spectrophotometer (Thermo Scientific, Waltham, MA, USA) and Qubit 2.0 fluorometer (Life Technologies, Carlsbad, CA, USA) were used to assess the purity and quantity of the DNA, respectively.

### Construction of Recombinant *cneI* Expression Plasmids

The autoinducer synthase gene, *cneI*, was amplified from the genome of SSMD04 by polymerase chain reaction (PCR) using the following primers: *cneI*-F-NcoI (5′-CCATGGCGATGTGTTCTGCAATTGAA-3′) and *cneI*-R-BamHI (5′-GGATCCTTGAGGTGGTTGAGCTGTGT-3′). The NcoI and BamHI restriction sites were underlined in the primer sequences. The stop codon was removed in the reverse primer design to incorporate a C-terminal His-tag into the recombinant protein. The PCR was performed on Veriti^®^ Thermal Cycler (Thermo Scientific, Waltham, MA, USA) with the following conditions: initial denaturation at 95°C for 3 min, followed by 30 cycles of denaturation at 95°C for 30 s, annealing at 49°C for 30 s, extension at 72°C for 1 min, and a final extension at 72°C for 7 min. Upon running agarose gel electrophoresis, the desired amplicon was extracted using Wizard^®^ SV Gel and PCR Clean-Up System (Promega, USA). The purified amplicon was ligated into a pGEMT-Easy vector (Promega, USA) per manufacturer’s instructions, followed by transformation into competent *E. coli* DH5α. The recombinant vector, designated pGEMT-Easy_*cneI*, was purified and linearized with both NcoI and BamHI enzymes (NEB, USA). This insert was then purified and ligated into pET28a (Novagen, Germany) that was linearized with the same restriction enzymes. The resulting recombinant plasmid was designated pET28a_*cneI*. The sequence of both recombinant plasmids was verified by automated Sanger sequencing.

### Nucleotide Sequence and Bioinformatics Analysis

The nucleotide sequence of *cneI* was retrieved from Rapid Annotation using Subsystem Technology (RAST) (Aziz et al., 2012) using “Genome Browser” function. This sequence was compared with GenBank databases using BLASTP program^1^ with default parameters. Ten LuxI homologs with the highest identity, omitting redundant or ambiguous sequences, were selected from the protein database. The sequences were aligned with ClustalW using MEGA 6.0 (Tamura et al., 2013) and a Maximum Likelihood phylogenetic tree was constructed from the aligned sequences using 1,000 bootstrap replications. The alignment of the LuxI homologs was presented using ESPript (Robert and Gouet, 2014).

### Detection of AHL Production in *E. coli* Expressing *cneI*

The recombinant plasmid pET-28a_*cneI* was transformed into *E. coli* BL21(DE3)pLysS cells and the transformants were selected using LB plates supplemented with kanamycin and chloramphenicol. Verification of transformants was performed using colony PCR followed by automated Sanger sequencing. The desired transformant was tested against an AHL biosensor *Chromobacterium violaceum* CV026 (McClean et al., 1997). The positive and negative controls used were *E. carotovora* GS101 and *E. carotovora* PNP22 (Jones et al., 1993), respectively. *E. coli* transformed with pET-28a alone was also used as a negative control.

### Heterologous Expression of CneI and His-Tagged Protein Purification

*Escherichia coli* BL21(DE3)pLysS harboring pET-28a_*cneI* was grown to an OD600 reading of 0.5–0.6. The cultures were then supplemented with either 0.5 or 1.0 mM of isopropyl β-D-1-thiogalactopyranoside (IPTG) and further incubated for 8 h with shaking at 25°C. The cultures were subsequently used for AHL extraction or protein purification. For protein purification, the cells were collected via centrifugation at 10,000 rpm and lysed using BugBuster^TM^ Protein Extraction Reagent supplemented with protease inhibitors (Novagen, Germany). His-tagged protein was subsequently purified from the cell lysate using Ni-NTA Fast Start Kit (Qiagen, Germany) according to manufacturer’s protocol.

### Extraction of AHL and its Identification by Triple Quadrupole LC/MS

*N*-acyl homoserine lactone extraction and identification by triple quadrupole LC/MS was performed as described previously (Tan et al., 2015). In brief, a cell-free supernatant of *E. coli* BL21(DE3)pLysS expressing *cneI* was subjected to AHL extraction using an equal amount of acidified ethyl acetate (AEA) (0.1% v/v glacial acetic acid) twice. The dried extracts were finally reconstituted in 1 mL of acetonitrile (ACN), which was then analyzed by triple quadrupole LC/MS using precursor ion scan mode (Tan et al., 2015).

### Sodium Dodecyl Sulfate Polyacrylamide Gel Electrophoresis (SDS-PAGE)

The induced bacterial cultures were harvested and the cell pellets were collected by centrifugation. The pellets were then resuspended in BugBusterTM Protein Extraction Reagent supplemented with protease inhibitors (Novagen, Germany). The samples were heated at 95°C for 3 min prior to loading into 12.5% (w/v) polyacrylamide gel electrophoresis system (PAGE, Bio-Rad, USA) in the presence of sodium dodecyl sulfate (SDS). The gel was subsequently stained with Coomassie Brilliant Blue R-250 (CBB; Bio-Rad, USA) for visualization of protein bands.

## Results

**FIGURE 1:**
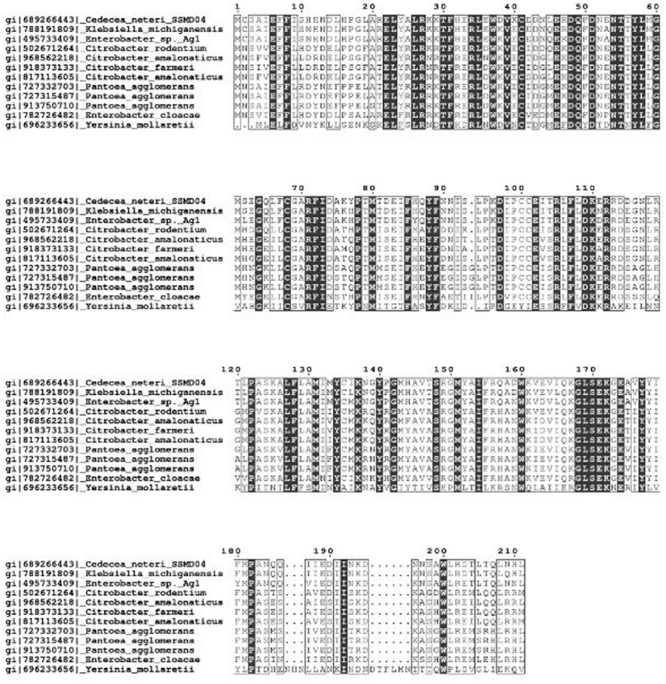
**Multiple sequence alignment of CneI with other LuxI homologs that share the highest similarity score in GenBank database.** Identical residues are shown as vertically filled bars, and the conserved residues are shown as the unfilled bars. Residues 27, 31, 37, 46, 48, 51, 71, 85, 102, and 105 are the invariant residues in LuxI homologs.

The protein sequence of CneI was searched against the NCBI database using BLAST and it was found to have a high degree of similarity with several protein sequences. It shows the highest similarity (94%) with LuxI homolog from *Klebsiella michiganensis*. These LuxI homologs are known to share high homology, particularly the 10 invariant residues, characteristics of LuxI homologs (Fuqua et al., 2001) (**Figure 1**).

**FIGURE 2:**
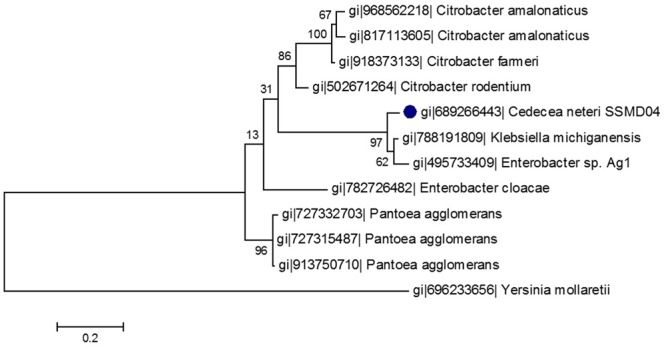
**Phylogenetic tree of CneI (solid black circle) and other homologous proteins of high similarity.** This maximum likelihood tree was constructed using Jones-Taylor-Thornton (JTT) model with gamma distribution. Thousand bootstrap replication was used and the bootstrap values are shown at the nodes. The scale represents the number of substitutions per amino acid position. The LuxI homolog of *Yersinia mollaretii* was used as an outgroup.

A phylogenetic tree was constructed from the 10 LuxI homologs that share the highest sequence similarity with CneI. As was shown in **Figure 2**, CneI is most closely related to LuxI homologs of *K. michiganensis* and *Enterobacter* sp. Ag1 instead of *Citrobacter rodentium* as previously thought (Tan et al., 2015). LuxI homologs from the *Citrobacter* genus seem to belong to a group of its own as they form a cluster, suggesting conservation of LuxI homolog sequence in this genus.

**FIGURE 3:**
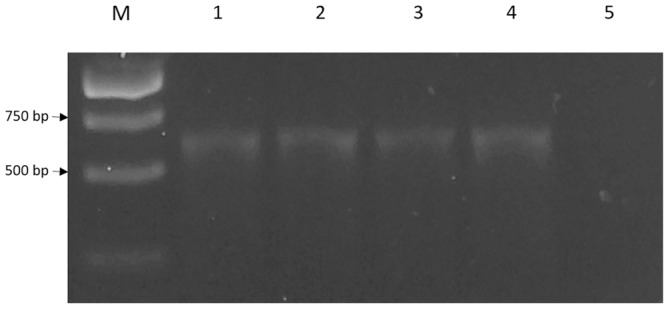
**The 636 bp *cneI* fragments amplified from genomic DNA of strain SSMD4.** Lane M represents 1 kb DNA ladder. The PCR reaction was performed in replicates (lanes 1–4). Lane 5, negative control.

The 636 bp *cneI* was identified from the whole genome sequence (Chan et al., 2014) and amplified from the genomic DNA of strain SSMD04 by PCR (**Figure 3**) and cloned into an expression vector, pET28a. This recombinant plasmid was named pET28a_*cneI*. This gene was cloned upstream of a 6x His-tag sequence under the control of a T7 promoter. Expression of *cneI* in *E. coli* BL21(DE3)pLysS was induced by IPTG. Two concentrations of IPTG, 0.5 and 1.0 mM, were used for induction but 1.0 mM of IPTG was later chosen as the optimum concentration (data not shown).

**FIGURE 4:**
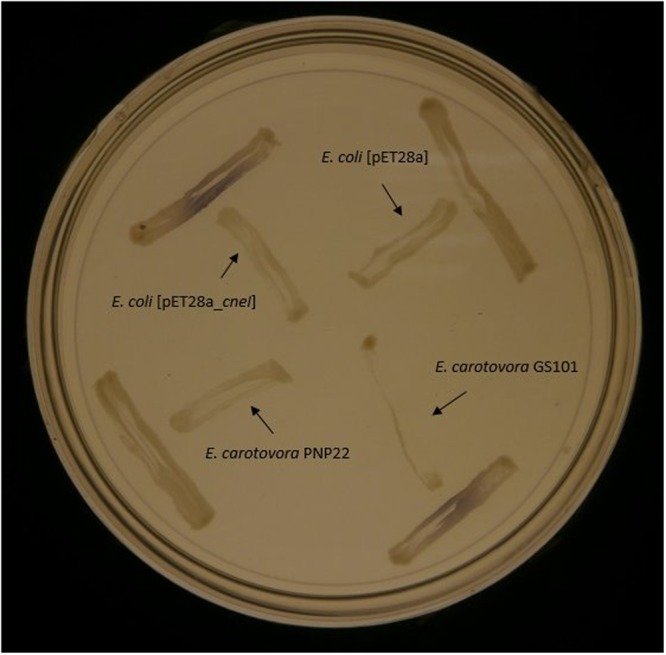
**Screening of AHL synthesis by CneI in *E. coli* BL21(DE3)pLysS using biosensor *C. violaceum* CV026.** The biosensor was streaked perpendicularly against all tested strains at the peripheral of the plate. Purple pigmentation produced by the biosensor indicates AHL production by the tested strain. *E. carotovora* PNP22 and *E. carotovora* GS101 act as positive and negative controls, respectively.

*E. coli* BL21(DE3)pLysS harboring pET-28a_*cneI* was shown to induce purple pigmentation in the biosensor *C. violaceum* CV026, while *E. coli* BL21(DE3)pLysS harboring pET-28a alone did not activate violacein production in CV026. This strongly suggests the role of *cneI* in the synthesis of short-chain AHL (**Figure 4**).

**FIGURE 5:**
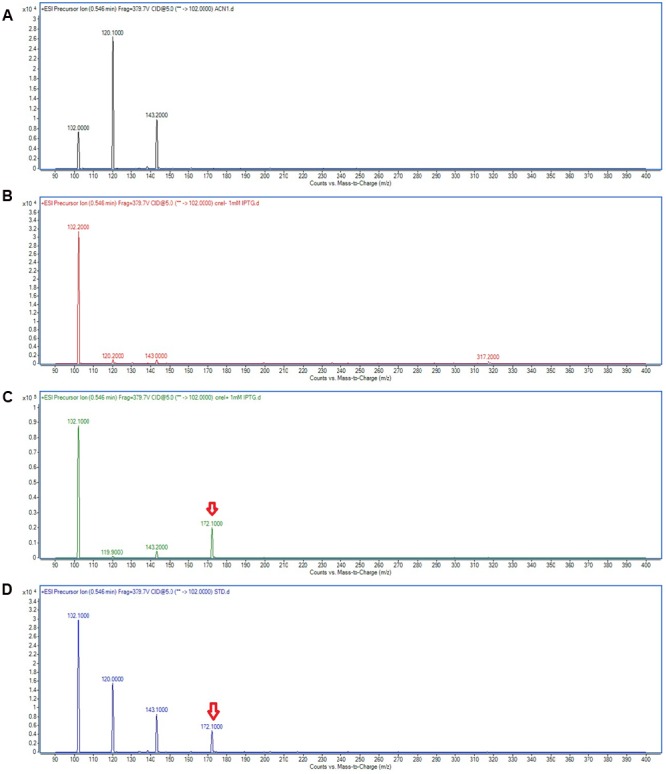
**Mass spectra showing the AHL profile of spent culture supernatant of *E. coli* BL21(DE3)pLysS harboring pET28a_*cneI*.** ACN **(A)** was used as blank. The EIC spectra of *E. coli* with pET_28a alone **(B)** and with pET28a_*cneI* **(C)** were compared with that of synthetic AHL, C4-HSL **(D)** at the same retention time. The detection of the peaks with *m/z* 172.100 signify the presence of C4-HSL as shown by arrows.

Triple quadrupole LC/MS was then used to determine the AHL profile of *E. coli* harboring pET28a_*cneI* after induction with IPTG. The cells were induced with 0.5 and 1.0 mM of IPTG and grown for 8 h after induction at 25°C. The extracted-ion chromatogram (EIC) showed a peak with *m/z* 172.0 at the same retention time (0.546 min) with that of the synthetic *N*-butyryl-homoserine lactone (C4-HSL), suggesting the presence of C4-HSL in the organic extract of the induced *E. coli*. The same result was observed when two different concentrations of IPTG (0.5 and 1.0 mM) were used for induction. However, no AHL was detected in the spent culture supernatant of *E. coli* harboring pET28a alone (**Figure 5**). The structure of the AHL produced was later confirmed through the examination of the product ions of the peak found in EIC. The presence of the peak (*m/z* 102.100) indicates the presence of a homoserine lactone ring moiety, the core structure of AHL molecule.

**FIGURE 6:**
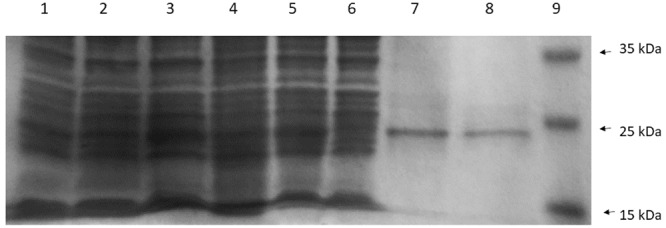
**The sodium dodecyl sulfate polyacrylamide gel electrophoresis (SDS-PAGE) profile of CneI overexpression (Lanes 1–5) and purification (Lanes 6–8) from *E. coli* BL21(DE3)pLysS.** Lane 1: Cell lysate of *E. coli* BL21(DE3)pLysS. Lanes 2 and 3: Cell lysate of *E. coli* BL21(DE3)pLysS transformed with empty pET28a with and without IPTG induction, respectively. Lanes 4 and 5: Cell lysates of *E. coli* BL21(DE3)pLysS harboring pET28a_*cneI* with and without induction, respectively. Lane 6: Flowthrough fraction. Lane 7: Wash fraction. Lane 8: Eluted fraction. Lane 9: PageRuler prestained protein ladder in kiloDalton (kDa). (Thermo Scientific, Waltham, MA, USA).

The recombinant His-tagged CneI protein was later purified and examined by SDS-PAGE (**Figure 6**). Despite overexpression, the protein profile did not show a very apparent CneI band (Lane 4) compared to the ones without it (Lanes 1–3, and Lane 5). Nevertheless, subsequent purification using nickel-based affinity column showed that the recombinant protein of CneI has a molecular weight of approximately 25 kDa.

## Discussion

*N*-acyl homoserine lactone-type QS was first discovered in a marine bacterium, *A. fischeri* in the regulation of bioluminescence (Hastings and Nealson, 1977; Nealson and Hastings, 1979). Decades of research have found a link between QS and many other phenotypes. Virulence and food spoilage traits (Thomson et al., 2000; Weeks et al., 2010; Skandamis and Nychas, 2012) are common QS-controlled phenotypes found in *Pseudomonas aeruginosa* (Whiteley et al., 1999), *Serratia proteamaculans* (Christensen et al., 2003), *S. liquefaciens* (Riedel et al., 2001) and *E. carotovora* (Pirhonen et al., 1993). A well-exemplified characteristic is the formation of biofilm. Many microbial species encase their colonies within an extracellular matrix called biofilm, which provides resistance toward antimicrobial substances as well as cleaning agents, making them hard to be eradicated (Costerton et al., 1995; Skandamis and Nychas, 2012), thus elevating their virulence and food spoilage potential. It is known that many bacteria such as *P. aeruginosa, Aeromonas hydrophila, Burkholderia cepacia, S. liquefaciens*, and *Pantoea stewartii* employ AHL-type QS as a mean of regulation of biofilm formation (Huber et al., 2001; Lynch et al., 2002; Labbate et al., 2004; Koutsoudis et al., 2006).

Besides biofilm formation, many genes related to pathogenicity and food spoilage are cell density-dependent in their expressions, such as virulence determinants, proteolytic or lipolytic enzymes, and biosurfactant. These genes share a similarity in that they synthesize exo-products, which benefit from cell density-dependent regulation. This is because a timed expression of a gene leads to a high production of such products, which inadvertently amplifies the effects brought by the secreted products. In addition, a favorable external environment facilitates the activation of QS circuit, hence, the secretion of exo-products. However, there was an alternative explanation for bacterial cell-to-cell signaling based on this foundation, despite not being the most commonly accepted. This hypothesis, known as diffusion sensing, suggests that bacterial cells use the concentration of signaling molecules to gauge the rate of diffusion of exo-products instead of population density. Therefore, a high extracellular concentration of signaling molecules signifies a favorable environment for the secretion of exo-products as the rate of diffusion is low (Redfield, 2002).

Similar to strain SSMD04, some aforementioned bacteria produce C4-HSL as the signaling molecule as well. *A. hydrophila*, for example, was reported to possess a *luxI* homolog termed *ahyI*, which synthesizes C4-HSL. It has been found that C4-HSL in this food poisoning species is critical in its biofilm formation and extracellular protease production (Swift et al., 1999; Lynch et al., 2002). Both of these traits are relevant to not only to its pathogenicity but also its food spoilage potential. Although QS in *A. hydrophila* has not been shown explicitly to affect its food spoilage trait, we believe it is likely due to the fact that C4-HSL from this bacterium has frequently been detected in food sources such as aerobically-chilled stored ground beef and commercial bean sprouts. It was reported that C4-HSL was believed to be responsible for food spoilage (Skandamis and Nychas, 2012). Therefore, the discovery of AHL-type QS in strain SSMD04 may suggest the same association in this bacterium.

Apart from that, a close relative of the *Cedecea* genus, *S. liquefaciens*, also employs C4-HSL as its signaling molecule. Mutant deficient of QS regulation in *S. liquefaciens* was found to lose the ability to produce biosurfactant, which is responsible for the swarming motility in *Serratia* spp. (Lindum et al., 1998). However, QS-regulated genes are not necessarily genes that code for the exo-products. In *S. liquefaciens*, QS also positively regulates the expression of *lipBCD*, which encode Lip exporter, a type I secretion protein that mediates the secretion of metalloprotease, lipase, and S-layer protein. A loss in QS regulation led to reduced secretion of metalloprotease and S-layer protein (Riedel et al., 2001). We, therefore, hypothesize that QS in strain SSMD04 is involved in the regulation of exo-products expression or secretion, which is very likely to cause an altered phenotype in its virulence or food spoilage traits. However, this hypothesis is yet to be validated.

The LuxI homolog found in strain SSMD04 was previously thought to be a homolog of CroI found in *C. rodentium* (Coulthurst et al., 2007) through BLAST search against NCBI database. However, the addition of new genome sequences to the GenBank database changed our perspective. We found another sequence from *K. michiganensis* which showed a much higher sequence similarity to CneI. *K. michiganensis* is a new member of the genus *Klebsiella* first reported by Saha et al. (2013). However, it was not until 2 years later that the genome was sequenced and enabled us to find its LuxI homolog.

The multiple sequence alignment and the phylogenetic tree constructed (**Figures 1** and **2**) illustrated a high degree of homology and conserved regions among several members of LuxI family. All the strains shared the 10 invariant amino acids which are characteristics of LuxI homologs. This strongly indicates a low rate of random mutation for these autoinducer synthase genes even though they are from different genera. It also shows that these proteobacteria share similar basic QS mechanism and gene regulation in AHL synthesis although they are responsible for different target genes. In fact, random and site-directed mutagenesis demonstrated that the amino acids are important in the binding of acyl substrates to QS signal generators (Parsek et al., 1997). Out of the 10 LuxI homologs that share the highest sequence similarities with CneI, only a few of the QS circuits have known regulatory functions. *C. rodentium*, for example, uses QS to regulate its surface attachment as its QS mutant adheres less well compared to its wild type counterpart (Coulthurst et al., 2007). On the other hand, gall-forming *Pantoea agglomerans* utilizes QS system to regulate its gall formation on plants, as the loss of QS regulation led to reduction in the gall size (Chalupowicz et al., 2008). Interestingly, LuxI homologs in both *C. rodentium* and *P. agglomerans* produce C4-HSL as the major AHL. Other bacterial species have been reported to possess LuxIR homologs but their regulatory role remains unknown.

The study of QS has been of great interest in that QS has been shown to be a suitable approach in the interruption of the regulated phenotype such as virulence (Rutherford and Bassler, 2012; Singh, 2015). Over the years, a large number of LuxI homologs have been found (Case et al., 2008), but it is not possible to predict the nature of AHL(s) produced by a given LuxI homolog based on its sequence alone. Expressing the *luxI* homolog in *E. coli* or comparing the wild type parent against a corresponding *luxI* mutant has been the only option in order to elucidate the type of AHL(s) produced by a given LuxI (Ortori et al., 2007). In this study, we have successfully cloned *cneI* and expressed its protein, CneI, in an *E. coli* host. *E. coli* does not produce AHL despite possessing an orphan LuxR, the SdiA (Kanamaru et al., 2000), therefore making it an ideal host to characterize CneI. As many LuxI type proteins synthesize more than one type of AHLs (Parsek et al., 1999), it is worth noting that only C4-HSL was produced by strain SSMD04, indicating the specificity of a single AHL in regulating physiological processes and virulence of the bacterium.

Expression of *cneI* in *E. coli* leads to the synthesis of C4-HSL, strongly suggests that CneI is indeed the AHL synthase of strain SSMD04. The expression level of *cneI* under the control of T7 promoter was high such that sufficient C4-HSL was produced to activate the production of purple violacein by the biosensor *C. violaceum* CV026 without IPTG induction (**Figure 4**). Purification of CneI and subsequent SDS-PAGE analysis showed that CneI was highly expressed with a molecular weight of approximately 25 kDa, in agreement with the prediction by bioinformatics tools. The expected molecular weight of CneI is 24.9 kDa as predicted from Expasy software^2^. Purification of CneI using Ni-NTA column also enables the protein to be found in soluble fraction, hence, a possible large scale purification of CneI for future work in protein characterization.

This study is the first documentation of cloning and molecular characterization of *luxI* homolog from *C. neteri*. The study on the autoinducer synthase gene, *cneI*, helps in providing further information in the elucidation of QS in *C. neteri*, as well as paving the way for future studies on the regulatory role of QS in this bacterium. This gene is also a suitable candidate for the interception of QS in this bacterium, providing ways of further research on the regulatory network of QS as well as diversifying the knowledge of the possible roles played by QS. On a final note, this work makes it possible to perform a genome-wide comparative transcriptomics by knocking out *cneI* for further investigations.

## Author Contributions

K-HT, K-YH, J-YT, and W-FY performed the experiments and analyzed the data. K-HT and K-YH prepared the draft and K-GC approved the final draft. K-GC conceived the ideas, supervised, applied for funding and monitored the entire project.

## Conflict of Interest Statement

The authors declare that the research was conducted in the absence of any commercial or financial relationships that could be construed as a potential conflict of interest.

## Footnotes

1 http://www.ncbi.nlm.nih.gov/

2 http://web.expasy.org
